# A Pharmacologic Approach Against Glioblastoma—A Synergistic Combination of a Quinoxaline-Based and a PI3K/mTOR Dual Inhibitor

**DOI:** 10.3390/ijms26136392

**Published:** 2025-07-02

**Authors:** Vitória Santório de São José, Bruno Marques Vieira, Camila Saggioro de Figueiredo, Luis Gabriel Valdivieso Gelves, Vivaldo Moura Neto, Lídia Moreira Lima

**Affiliations:** 1 Laboratório de Avaliação e Síntese de Substâncias Bioativas (LASSBio^®^), Instituto Nacional de Ciência e Tecnologia de Fármacos e Medicamentos (INCT-INOFAR), Universidade Federal do Rio de Janeiro, Rio de Janeiro 21941-902, Brazil; vitoriassjose@gmail.com (V.S.d.S.J.); luisga011@hotmail.com (L.G.V.G.); 2 Programa de Pós-Graduação em Farmacologia e Química Medicinal, Instituto de Ciências Biomédicas, Universidade Federal do Rio de Janeiro, Rio de Janeiro 21941-902, Brazil; 3 Laboratório de Biomedicina do Cérebro, Instituto Estadual do Cérebro Paulo Niemeyer (IECPN), Rio de Janeiro 20231-092, Brazil; brunomarquesv@gmail.com (B.M.V.); vivaldomouraneto@gmail.com (V.M.N.); 4 Laboratório de Medicina Experimental e Saúde, Instituto Oswaldo Cruz, Fiocruz, Rio de Janeiro 21040-360, Brazil; 5 Laboratório de Neuropatologia, Instituto Estadual do Cérebro Paulo Niemeyer (IECPN), Rio de Janeiro 20231-092, Brazil; camila.saggioro06@gmail.com

**Keywords:** glioblastoma, EGFR, PI3K, mTOR, synergistic effect, LASSBio-1971, Gedatolisib

## Abstract

Glioblastoma (GB) is the most common malignant primary CNS tumor with a fast-growing and invasive profile. As a result of the poor prognosis and limited therapy available, glioblastoma shows a high mortality rate. Given the scarcity of effective chemotherapy options, multiple studies have explored the potential of tyrosine kinase inhibitors. To mitigate resistance and improve potency and selectivity, we proposed the combination of a potent irreversible epidermal growth factor receptor inhibitor—LASSBio-1971—and a potent phosphatidylinositol-3-kinase/mammalian target of rapamycin dual inhibitor—Gedatolisib—through an in vitro phenotypic study using five human GB lines. Here, we aimed to establish the cytotoxic potency, selectivity, and effect on proliferation, apoptosis, migration, and the cell cycle. Our data showed the cytotoxic potency of Gedatolisib and LASSBio-1971 and improved selectivity in the GB cell lines. They highlighted the synergistic response from their combination and its impact on migration reduction, G0/G1 cell cycle arrest, GB cytotoxicity, and apoptosis-inducing effects for different GB cell lines. The drug combination studies in phenotypic in vitro models made it possible to suggest a new potential treatment for glioblastoma that justifies further safety in in vivo phases of preclinical trials with the combination.

## 1. Introduction

Glioblastoma (GB) is an aggressive and highly malignant primary brain tumor with a poor prognosis and limited treatment options, primarily due to its invasiveness, resistance to therapy, and genetic heterogeneity. Despite advancements in surgical resection, radiotherapy, and chemotherapy, the median survival remains approximately 15 months [1,2]. According to the 2021 WHO Classification of Tumors of the Central Nervous System, GB is now exclusively defined as an IDH-wildtype astrocytic tumor. IDH-mutant diffuse astrocytomas, including grade 4 forms, are categorized separately and are no longer referred to as GB. GBs occur predominantly in older adults and are associated with aggressive behavior and poor prognosis due to genetic alterations such as epidermal growth factor receptor (EGFR) amplification and Phosphatase and Tensin Homolog (PTEN) loss [3].

The currently available therapeutic strategy has limitations, particularly regarding the low potency, efficacy, and permeability of the blood–brain barrier (BBB) of standard chemotherapy. This standard for GB, established in 2005 [4], remains unchanged, showing only minimal variations [5], namely, chemotherapy with temozolomide (TMZ) alone or combined with radiotherapy for patients with or without the methylated O-6-methylguanine-DNA methyltransferase (MGMT) promoter, respectively [6]. More than 50% of patients do not respond to TMZ [7], resulting in therapeutic inefficacy.

One of the key drivers of glioblastoma progression is the dysregulation of several critical signaling pathways, including those involving the EGFR and the phosphatidylinositol-3-kinase (PI3K)/mammalian target of rapamycin (mTOR) axis [8,9]. The EGFR pathway, often mutated or overexpressed in glioblastoma, plays a significant role in cell proliferation, survival, and invasion [9,10]. Mutations in the EGFR gene, such as the EGFRvIII variant, lead to constant receptor activation and downstream signaling through the phosphatidylinositol-3-kinase (PI3K)/mammalian target of rapamycin (mTOR) pathway, promoting tumor growth and therapy resistance [11]. Similarly, aberrations in the PI3K/mTOR pathway, often due to loss of the tumor suppressor PTEN, further contribute to the malignancy of glioblastoma, facilitating uncontrolled cell growth and survival [12].

Monotherapy with agents targeting these pathways has shown limited efficacy, largely due to GB’s complex molecular landscape and ability to activate compensatory mechanisms [8,12]. Consequently, combining inhibitors that target multiple pathways simultaneously has emerged as a promising strategy. Polytherapy, which involves the concurrent use of multiple drugs, can potentially overcome resistance mechanisms that monotherapies cannot address [13,14]. By inhibiting both EGFR and PI3K/mTOR pathways, this approach may reduce tumor cell viability more effectively and delay the development of resistance [15,16,17].

Given the limitations of current treatment modalities and the resilience of glioblastoma, it is essential to explore new therapeutic targets and combinations. This includes the development of novel inhibitors, such as the EGFR inhibitor LASSBio-1971 [18]. LASSBio-1971 inhibits different forms of EGFR, has a good membrane permeability that mimics the blood–brain barrier (BBB), and already displayed promising results in various tumor cell lines [18]. Thus, this study aims to evaluate polytherapy using LASSBio-1971 and the dual PI3K/mTOR inhibitor PKI-587 to bypass tumor resistance mechanisms, accessing the synergist effect and the mechanisms of action of the polytherapy by flanking different cellular pathways involved in GB biology.

This project was developed in parallel with a previously published study evaluating the combination of Osimertinib and Gedatolisib in glioblastoma cell lines [17]. While the experimental design is shared to ensure comparability, the current manuscript focuses on the novel EGFR inhibitor LASSBio-1971. Therefore, this study aimed to investigate the in vitro therapeutic potential of combining the EGFR inhibitor LASSBio-1971 and the PI3K/mTOR inhibitor PKI-587. We evaluated their cytotoxicity, synergy, impact on cell migration, apoptosis, cell cycle arrest, and BBB permeability using glioblastoma models.

## 2. Results

### 2.1. Selection of BBB-Permissive Inhibitors

**Figure 1:**
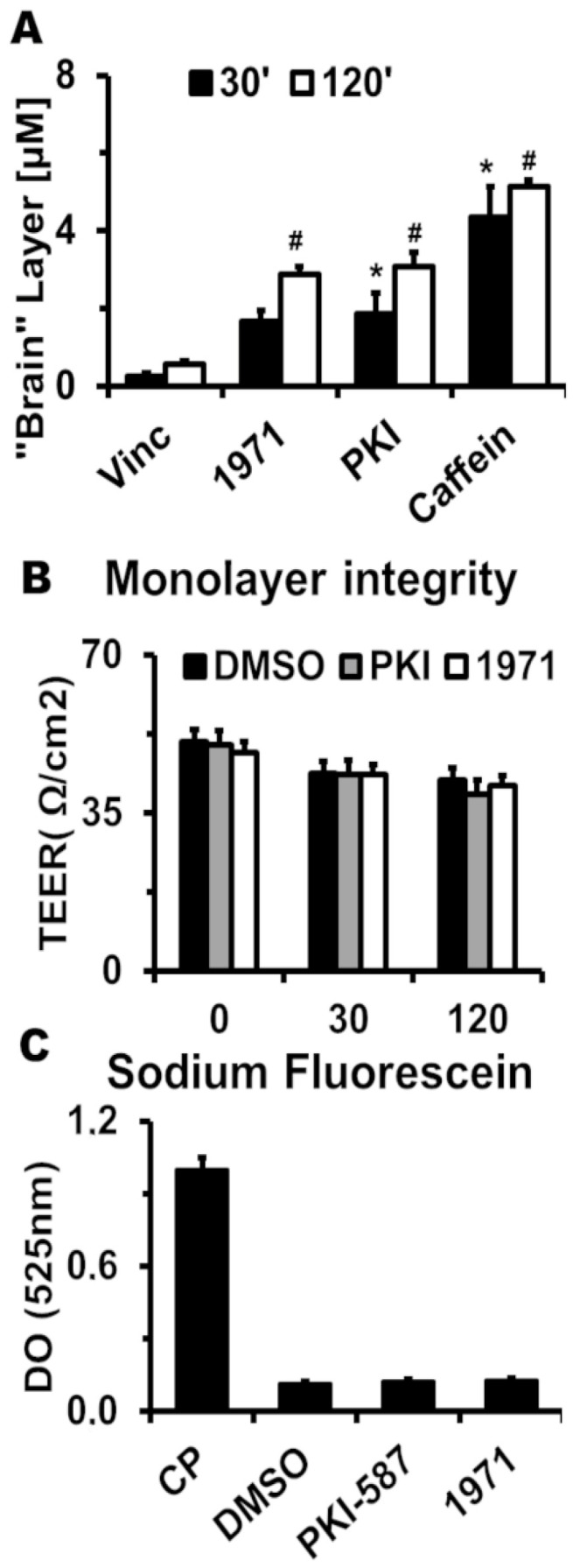
LASSBio-1971 and PKI-587 are BBB permeable. (**A**) Permeability prediction of LASSBio- 1971 and PKI-587, after 30 and 120 min, was based on HBMEC monolayer assay. Vincristine and caffeine were used as negative and positive control, respectively. (**B**) Transendothelial electrical resistance was used as a parameter of HBMEC monolayer integrity after 30 and 120 min of incubation with vehicles (DMSO), PKI-587 and LASSBio-1971, respectively. (**C**) Sodium fluorescein assay was used to assess paracelular transport of HBMEC monolayer. CP: positive control. #, significant differences between the indicated group and the “30′” group (*p* < 0.05) in ANOVA. *, significant differences between the indicated group and the “DMSO” group (*p* < 0.05) in ANOVA. *n* = 6 (2 biological × 3 technical replicates) for all groups.

To evaluate the BBB permeability from LASSBio-1971 and PKI-587, we used an HBMEC monolayer in a transwell system. As seen in Figure 1A, PKI-587 (PKI group) was found at 30 and 120 min in the lower chamber (brain layer), in a significantly higher concentration compared to the vincristine (Vinc group) negative control. For LASSBio-1971 (1971 group), the statistical increase, compared to the Vinc group, was observed only at 120 min. Caffeine was used as a positive control, as caffeine is permeable to the HBMEC monolayer. The integrity of the HBMEC monolayer after the treatments were accessed by TEER (Figure 1B) and sodium fluorescein translocation (Figure 1C). In both cases, the treatment with PKI-587 or LASSBio-1971 did not decrease the TEER or increase the sodium fluorescein concentration in the lower chamber, compared to the “DMSO” control group. The CP group in Figure 1C shows the maximum translocation in the absence of the HBMEC monolayer.

Next, we evaluated the cytotoxic effect of the inhibitors, accessing the maximum effect (%Emax), CC50, and selective index (SI), at 24 and 72 h, in five different GB cell lines (GBM02, GBM03, GBM95, T98 G, and A172). We confirmed that all the cell lines were GB by genotyping the codon 132 of IDH1 by Sanger sequencing. All the cell lines were IDH wild-type (Supplementary Figure S1).

In the MTT assay, the PKI-587 monotherapy exhibited significant cytotoxic effects on glioblastoma cells (Table 1). After 72 h, its Emax exceeded 95.5%, and it demonstrated potency greater than 0.03 μM across all glioblastoma cell lines. However, the cytotoxic response observed at 24 h was considerably lower in both potency and maximal effect on cell viability, indicating a time-dependent response (Table 1; Supplementary Figure S2). This effect was observed in all cell lines, with a more pronounced impact on GBM02, GBM03, and GBM95.

As previously reported in [17], the cytotoxic profile of PKI-587 was established using a series of in vitro assays performed in parallel with the present study. These data are reused here to allow a direct comparison with LASSBio-1971 under identical experimental conditions. The LASSBio-1971 monotherapy demonstrated cytotoxic potency, with values greater than 2.3 μM (T98G) and less than 17.17 μM (GBM03), along with Emax values exceeding 85.4% (GBM03). Similar to PKI-587, the cytotoxic response of LASSBio-1971 was time-dependent, showing a reduction in Emax and an increase in CC50 after 72 h of incubation (Table 1).

Finally, the SI of the inhibitors was evaluated, determined by the ratio between the cytotoxic potency observed in astrocytes and the GB cell lines, using the MTT assay after 72 h of incubation. As shown in Table 1, PKI-587 demonstrated high selectivity, ranging from 51 (A172) to 933 (GBM95) more cytotoxic to tumor cells than ASTRh. LASSBio-1971 also showed selectivity, ranging from 3.05 (GBM03) to 5.45 (GBM95).

### 2.2. Synergistic Effect of LASSBio-1971 and PKI-587 Combination

**Figure 2:**
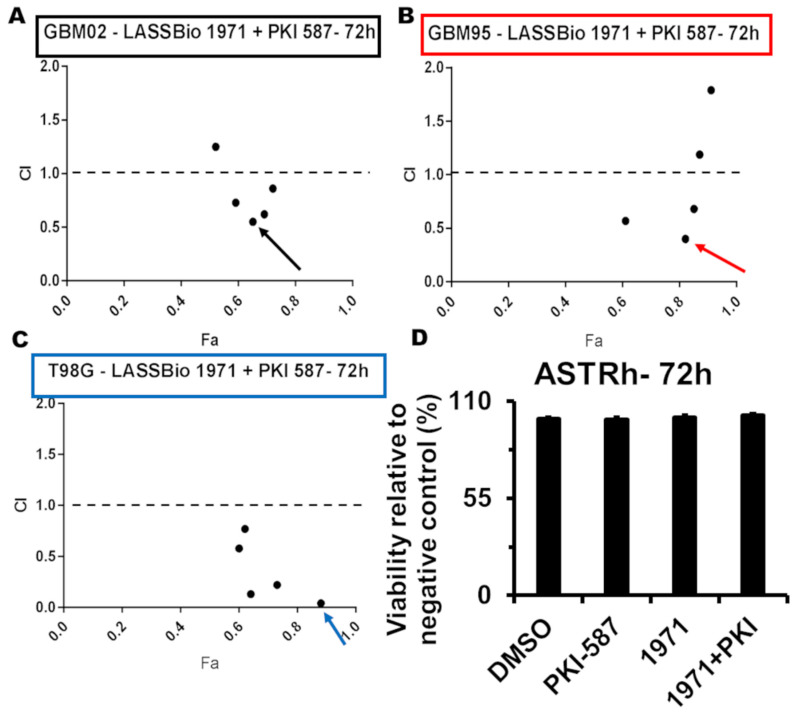
Synergistic effect of triple EGFR/PI3K/mTOR inhibition. GBM02 (**A**), GBM95 (**B**), and T98G (**C**) fraction affected/combination-index plot (CI values calculated using CompuSyn version 1.0). The best combination score was indicated by a black arrow (GBM02), a red arrow (GBM95), or a blue arrow (T98G). Viability of human astrocytes (**D**) incubated with 3 μM LASSBio-1971 and 0.0155 μM PKI-587 (red arrow in (**B**)), isolated or in combination. Values are means ± SEM. *n* = 6 (2 biological × 3 technical replicates) for all groups.

Here we focus on three cell lines to evaluate the combination mechanisms. The potential synergism from the combination of LASSBio-1971 and PKI-587 was studied using the Chou–Talalay method [19]. In Figure 2, we see the synergistic effect of the combinations on GBM 02 (A), GBM95 (B), and T98G (C) cell lines at 72 h. The combinations were selected based on the lowest CI and the highest Fa as follows: 7 µM LASSBio-1971 and 0.093 µM PKI-587 (GBM02), 3 µM LASSBio-1971 and 0.0155 µM PKI-587 (GBM95), and 1.75 µM LASSBio-1971 and 0.047 µM PKI-587 (T98G). In Figure 2D, we see that ASTRh did not lose viability with the combination (3 µM LASBio-1971 and 0.0155 µM PKI-587). Those initial in vitro results indicate that simultaneous EGFR/PI3K/mTOR inhibition with PKI-587 and LASSBio-1971 is a safe and effective strategy for GB therapy. Full CI values calculated by CompuSyn across multiple dose levels and Fa for all cell lines are summarized. These data support the consistent synergistic interaction observed between LASSBio-1971 and PKI-587 in GBM02, GBM95, and T98G cells.

The combination reduces migration, leading GB cell lines to G0/G1 arrest in vitro.

**Figure 3:**
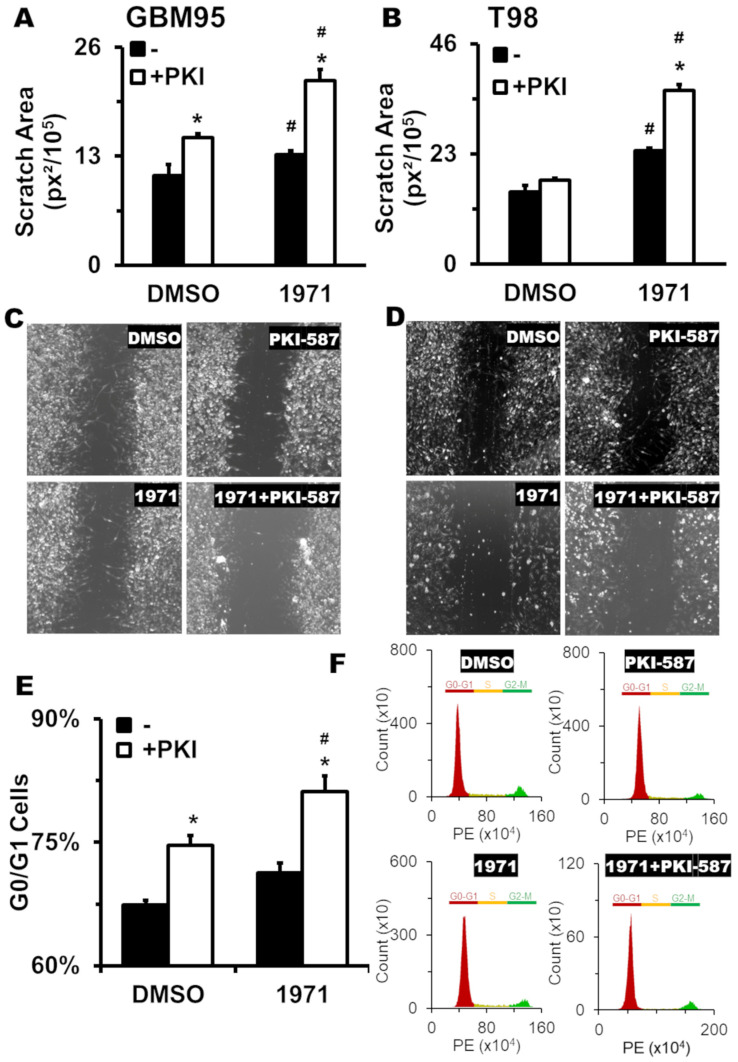
GB treatment impairs migration and cell cycle. GBM95 (**A**,**C**) and T98G (**B**,**D**) cells were treated with LASSBio-1971, PKI-587, or the combination in a scratch wound assay. Images in panels (**C**,**D**) show representative wound healing responses after 24 h. The images for the DMSO and PKI-587 conditions were reused from [17], as these controls were performed under the same experimental conditions and cell lines. Images for the 1971 and 1971 + PKI-587 groups were captured from the same experimental field but at a higher magnification (400×) to highlight morphological differences. The scratch area (**A**,**B**) was measured using photomicrography (**C**,**D**), obtained 24 h after the scratch. GBM95 cell cycle (**E**,**F**) was measured by PI incorporation in flow cytometry (**F**), and the 2n population was set as G1/G0 phase (**E**). #, significant differences between the indicated group and the “DMSO” group (*p* < 0.05) in ANOVA. *, significant differences between the indicated group and the “-PKI” respective group (*p* < 0.05) in ANOVA. *n* = 6 (2 biological × 3 technical replicates) for all groups. (**C**,**D**,**F**) display a representative analysis. This panel includes data previously published in [17], reused here for comparative purposes.

Next, focusing on two cell lines, we evaluated the effectiveness of the combination of LASSBio-1971 and PIK-587 in the migration response of the GBM95 and T98G cells. For this, we used the scratch wound assay. This method quantifies the open area from a GB monolayer after a scratch “wound”. For consistency, some images presented in Figure 3 were reused from our previous study [17], including the DMSO and PKI-587 controls, as the experiments were conducted in parallel using the same protocol and cell lines. Additionally, while the image fields remain the same, the panels corresponding to the LASSBio-1971 treatment were captured at higher magnification (400×) to emphasize morphological differences under this new condition. As shown in Figure 3, the LASSBio-1971 and PKI-587 in combination (Figure 3A,C for GBM95 and Figure 3B,D for T98G) significantly reduced cell migration, as shown by the increased wound area. Moreover, the combination shows increased inhibition compared to monotherapy. For the T98G cell line (Figure 3B,D), the PKI-587 monotherapy group did not show a statistical difference from the DMSO control group.

Further, we performed a flow cytometry assay to determine the cell cycle profile after therapy to identify if the EGFR/PI3K/mTOR inhibition reduces proliferation. As shown in Figure 3E,F, GBM95 cells displayed a G0/G1 arrest in the monotherapies (for both LASSBio-1971 and PKI-587), with an increased arrest in the combination, indicating a lower proliferative response than treatment with the drugs alone. The same G0/G1 arrest was found in T98G cells.

### 2.3. The Combination of LASSBio-1971 and PKI-587 Shows a Cytotoxic Profile and Induces In Vitro Apoptosis

**Figure 4:**
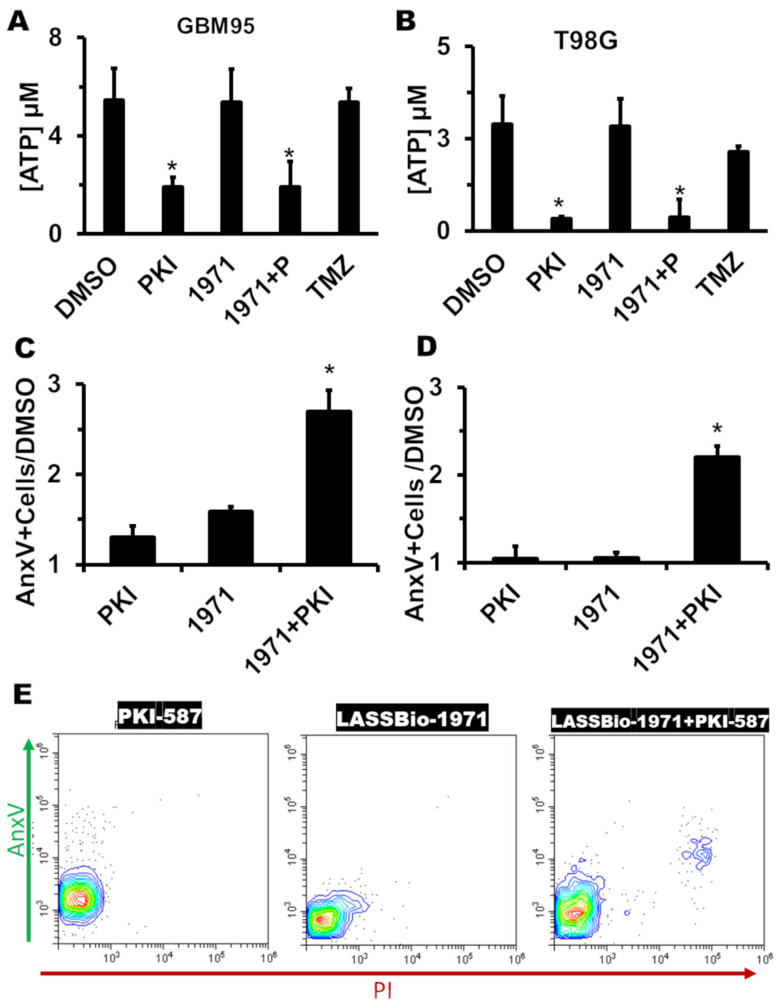
GB treatment induces apoptosis. GBM95 (**A**,**C**,**E**) and T98G (**B**,**D**) cells were treated with LASSBio-1971, PKI-587, or the combination. In (**A**,**B**), mono- and polytherapy reduced ATP levels, indicating a cytotoxic effect, while TMZ did not decrease ATP levels. Flow cytometry for Annexin V (AnxV) in GBM95 (**C**,**E**) and T98G (**D**) revealed increased apoptosis in treated groups. The DMSO control panel shown in (**E**) was reused from [17], as the control was performed under identical experimental conditions. *, significant differences between the indicated group and the “DMSO” group ((**A**,**B**) *p* < 0.05) in ANOVA or significant differences between the indicated groups ((**C**,**D**) *p* < 0.05) in ANOVA. Values are means ± SEM of two independent experiments in triplicate (**A**,**B**). *n* = 6 (2 biological × 3 technical replicates) for all groups (**C**,**D**). (**E**) displays a representative analysis of (**C**). This panel includes data previously published in [17], reused here for comparative purposes.

To determine whether the reduction in cell viability promoted by LASSBio-1971 and PKI-587 alone or in combination resulted from a cytotoxic or cytostatic action, we analyzed the effect of the inhibitors over intracellular ATP concentration after 72 h of incubation. DMSO was used as vehicle control, and TMZ was used as a cytostatic control [20,21,22,23]. This method indicates a cytostatic profile when ATP concentration is higher. Lower ATP concentration indicates a cytotoxic effect. Figure 4A,B show the cytotoxic effect of monotherapy with LASSBio-1971 and PKI-587 in the GBM95 and T98G cell lines, respectively. LASSBio-1971 by itself has a cytostatic effect but, in combination with PKI-587, shows lower ATP concentrations, indicating that blocking EGFR/PI3K/mTOR simultaneously induces a cytotoxic effect. To confirm this cytotoxic effect, we performed a flow cytometry assay to identify AnxV-positive cells (indicative of apoptosis) after 24 h of monotherapy or polytherapy. Figure 4C,E show a 2.5-fold increase in AnxV-positive GBM95 cells relative to the “DMSO” control. For the T98G cell line (Figure 4D), we see a 2.3-fold increase in AnxV-positive cells compared to the control. These results emphasize the benefits of blocking EGFR/PI3K/mTOR as a therapeutical alternative.

## 3. Discussion

Glioblastoma exhibits a high mortality rate and poor prognosis due to the tumor’s intrinsic characteristics: high proliferation, invasive capacity, privileged location, and inter- and intratumoral heterogeneity. Currently, the diagnosis is based on magnetic resonance imaging and computed tomography, along with histopathological and molecular characterizations obtained from biopsies during surgical resection [24]. The original histological findings corresponded to the GBM02, GBM03, and GBM95 cell lines showed vascular endothelial proliferation and cellular pleomorphism, consistent with the diagnosis of glioblastoma [25]. These findings build upon previous results from 2006, further confirming the molecular characterization (wild-type genotype for codon 132 of the IDH1 gene) of those cells. The selection of different glioblastoma cell lines, including commercially available ones, aimed to address the heterogeneity among patients diagnosed with GB.

For this study, LASSBio-1971 was selected from the LASSBio^®^ laboratory’s chemical library to be used as a prototype developed as an irreversible EGFR inhibitor, with proven potency in melanoma, breast, pancreatic tumors, and glioblastoma cell lines [18].

The importance of the EGFR signaling pathway in the pathogenesis of glioblastoma is described in the literature [26]. Activation of this pathway can be related to gain-of-function mutations, genomic amplification, chromosomal rearrangements, and autocrine activation. The effects of LASSBio-1971 are related to its irreversible inhibition of the EGF receptor, culminating in reduced activation of the PI3K/Protein kinase B (AKT)/mTOR and Phospholipase C Gamma pathways, which results in reduced angiogenesis, cell migration, invasion, proliferation, and metabolism [27]. Moreover, LASSBio-1971 can also irreversibly inhibit the EGFRvIII mutation, preventing the constitutive activation of the pathway [28]. However, we have found that LASSBio-1971 shows lower selectivity, compared to the PI3K/mTOR inhibitor PKI-587, in all cell lines. This can be due to the inhibition of other pathways that are activated in both tumor cells and non-tumoral astrocytes, such as mitogen-activated protein kinase (MAPK) interacting with protein kinases 1 and 2, which belong to the MAPK pathway and are related to the synthesis of the eukaryotic translation initiation factor 4G and the phosphorylation of eukaryotic translation initiation factor 4E [29]. This nonspecific inhibition resulting in lower selectivity can potentially be resolved by managing the therapeutic strategy, which will be addressed later.

The dual PI3K/mTOR inhibitor PKI-587 was selected for its in vitro potency against the α, β, γ, and δ isoforms of PI3K, mTORC1, and mTORC2 [30]. PKI-587 has already established potency and safety in clinical trials for solid and hematological tumors [31]. Monotherapy with PKI-587 reduces Akt phosphorylation, inhibiting the activation of the mTORC1 complex and leading to a reduction in the eukaryotic translation initiation factor 4E binding protein and the p70 subunit of S6 kinase, mitigating protein synthesis and cell growth, respectively [32]. The inhibition of mTORC1 is also related to reduced cell proliferation and autophagy, closely linked to tumor resistance mechanisms. The effect observed in glioblastoma cell lines in the presence of PKI-587 also encompasses the inhibition of the mTORC2 complex, which is associated with cytoskeletal organization, survival, and metabolism of tumor cells [32].

Chemotherapy-associated resistance is described for many EGFR/PI3K/mTOR inhibitors and includes acquired resistance-associated polymorphisms and tertiary mutations (extensively discussed in [33]), KRAS mutations, gene fusions, activation of PIM kinases, and increases in extracellular signal-regulated kinases (ERK)/MAPK signaling [33,34,35]. Although we cannot rule out the GB escape with these or other resistance mechanisms, our polytherapy successfully hindered GB in all the cell lines we explored.

The permeability assays across the BBB revealed that both compounds did not compromise the integrity of the HBMEC monolayer, assessed by TEER and paracellular transport. This indicates that the inhibitors were transported via active transport, which displays that PKI-587 and LASSBio-1971 exhibit permeability to the BBB. Although the permeability of LASSBio-1971 across the BBB has already been observed, it was in an artificial membrane model permeability model [36]. The permeability of PKI-587 was observed in an in vivo monkey model [37].

The observed potency of LASSBio-1971, after 72 h, was similar to PKI-587 potency, also showing an Emax close to 90%. For both, the responses were time-dependent, indicating a possible involvement of mechanisms dependent on the activation of transcription factors. The detailed mechanism of action of LASSBio-1971 is not yet fully understood, and thus further studies will be necessary to elucidate its action beyond the inhibition of the downstream EGFR pathway.

The PKI-587 monotherapy only reduced migration in the GBM95 cell line, unlike the LASSBio-1971, which hindered GB migration in GBM95 and T98G cell lines. The absence of reduced migration in T98G by PKI-587 can be justified by the influence of the β isoform, which is absent in GBM95 [17], as the expression of PI3Kβ is involved in resistance mechanisms to treatment with PKI-587 in the T98G cell line [38]. The PI3K/Akt pathway is directly related to the phosphorylation of the Ser9 of Glycogen Synthase Kinase 3 (GSK3β), culminating in its inhibition. GSK3β acts on the activation of Rho kinase family protein and Arf6, which are related to the formation of lamellipodia, which are crucial for cell migration [39].

Migration and invasion are key events in the metastatic process and begin with epithelial–mesenchymal transition (EMT). The inhibition of EGFR/PI3K/mTOR leads to reduced nuclear translocation of β-catenin, a co-activator for the transcription of genes related to invasion, proliferation, and maintenance of stem cell characteristics [40]. Thus, this inhibition is sufficient to reverse EMT. Our data demonstrates that, despite the different mechanisms of action of PKI-587 and LASSBio-1971, the combination is more effective in reducing migration than monotherapies, potentially mitigating the metastatic potential of the tumor in future preclinical and clinical trials.

Tumor progression is underpinned by the dysregulation of the cell cycle and inefficient regulation of the respective checkpoints [41]. Studies considering various tumor types have reinforced the importance of cell cycle suppression by inducing cell entrapment in G0/G1 through the inhibition of EGFR/MAPK [42,43,44,45,46] and PI3K/mTOR [47,48,49,50,51].

We chose the GBM95 cell line for the cell cycle/proliferation assay, as the T98G line exhibits polyploidy, and the quantification of the assay is based on diploid cells [52]. The combination of LASSBio-1971 and PKI-587 exhibited cell cycle entrapment effects and a consequent reduction in cell proliferation. The increase in cell entrapment in G0/G1 is accompanied by a reduction in the percentage of cells in the S- and G2/M phases, suggesting that the mechanism of action of the polytherapy involves the entrapment of cells in G1.

The characterization of cytotoxic or cytostatic action revealed a cytotoxic action of PKI-587 and a cytostatic effect for LASSBio-1971. The polytherapy presented a cytotoxic profile. The effect of cytotoxic antitumor agents, whether isolated or in combination, is described as a factor that promotes the reduction in tumor mass [53]. For this reason, the pharmacological combination strategy shows great potential to suppress tumor progression, compared to cytostatic drugs alone.

These observed cytotoxic effects may be explained by the induction of programmed cell death or necrosis. The polytherapy in the GBM95 and T98G cell lines exhibited an increase in the number of AnxV-positive cells, indicating an increase in cells at the early or late stage of apoptosis. Analyzing the distribution of the events observed in the flow cytometry graphs (dot plots), the data demonstrates that the mechanism of action of the combination involves an increase in apoptosis. The synergistic effects observed in both combinations regarding apoptosis—and not necrosis—are important to minimize adverse effects that may be observed in in vivo or clinical trials.

Compared to Osimertinib, as reported previously [17], LASSBio-1971 demonstrated similar or superior synergism when combined with PKI-587, particularly in terms of selectivity index and reduction in migration in GBM02 and T98G lines. While Osimertinib exhibited high potency, its lack of selectivity may limit its clinical translation. LASSBio-1971, although experimental, displayed promising cytotoxic selectivity profiles and might offer a broader therapeutic window, pending further pharmacokinetic evaluation.

Nonetheless, in clinical practice, the use of cytotoxic therapy may lead to adverse effects such as hyperuricemia, characteristic of tumor cell lysis. These adverse effects can be reduced or nullified by changes in cytotoxicity mechanisms, alterations in the dosing regimen, or by using adjuvant therapies that mitigate the adverse effects induced by cytotoxicity [54,55,56,57], making it safer to induce apoptosis in tumor cells.

Here we propose a combination of PKI-587 and LASSBio-1971 as a selective and effective therapy for GB, using an in-house alternative to EGFR inhibitors. Furthermore, the proposed combination allowed an increased potency and selectivity through dose optimization. The use of a combination of 3 µM of LASSBio-1971 and 0.0155 µM of PKI-587 was able to reduce viability by 82% in GBM95, while LASSBio-1971 monotherapy achieved the same efficacy at a concentration close to 18 µM. Thus, the combined therapy was responsible for reducing the concentration of LASSBio-1971 by 6-fold. In addition to the increase in cytotoxic potency, the combination was more selective, with no loss of viability in human astrocytes.

Thus, the combination of LASSBio-1971 and PKI-587 is a novel and strategically favorable strategy for GB therapy and is promising for initiating preclinical trials. This simultaneous inhibition of EGFR and PI3K/mTOR aims to block compensatory survival pathways and enhance cytotoxic efficacy. The current study was designed to assess the effect of a single treatment cycle; however, future studies will investigate repeated dosing to evaluate potential resistance mechanisms in surviving cell populations.

Regarding clinical relevance, PKI-587 has been evaluated in early phase clinical trials for solid tumors, with plasma concentrations in the sub-micromolar range deemed pharmacologically active and tolerable [31]. In contrast, LASSBio-1971 remains in the preclinical phase, and its maximum tolerated dose and full pharmacokinetic profile have yet to be determined. Therefore, although the in vitro concentrations used in our study fall within plausible translational ranges for PKI-587, further in vivo validation and toxicological evaluation will be essential for defining safe and effective dosing regimens, particularly for combination strategies.

Despite the promising in vitro results observed in this study, limitations should be acknowledged. The experiments were performed exclusively in cell culture models, which do not fully replicate the complexity of the tumor microenvironment, including immune components, stromal interactions, and in vivo pharmacokinetics. Furthermore, although both inhibitors crossed the in vitro BBB model, this finding requires confirmation in animal models that reflect the integrity and dynamics of the human BBB. Toxicological profiles and optimal dosing schedules remain unexplored, and repeated treatment cycles may reveal adaptive resistance mechanisms not captured here. Future steps will include orthotopic in vivo validation of efficacy and safety, along with pharmacokinetic and toxicity studies to support preclinical development of this combination strategy.

## 4. Methods

### 4.1. Ethics Statement and Glioblastoma Cell Cultures

This study was approved by the Ethics Committee at the Center for Health Sciences in the Federal University of Rio de Janeiro and by the Brazilian Ministry of Health Ethics Committee (CONEP Protocol No.2340). All experiments were performed under relevant guidelines and regulations. The GBM02, GBM03, and GBM95 glioblastoma cell lines were established and characterized in our laboratory [25,58]. The T98G cell line originated from the American Type Culture Collection. A172 glioblastoma cell lines were purchased from the European Collection of Authenticated Cell Cultures. All the GB cells were maintained in Dulbecco’s modified Eagle’s medium (DMEM), high glucose, supplemented with 10% fetal bovine serum (FBS) at 37 °C and in a controlled atmosphere containing 5% CO_2_. The adult human astrocyte cells (ASTRh) were isolated from patients selected for surgical treatment of temporal lobe epilepsy associated with hippocampus sclerosis at the Hospital Universitario Clementino Fraga Filho. All patients provided written informed consent to participate in the study, and the procedures agreed with the Brazilian Ministry of Health Ethics Committee (CONEP Protocol No.2340). The ASTRh cells were mantainedmaintained in DMEM, high glucose, supplemented with 10% FBS at 37 °C and in a controlled atmosphere containing 5% CO_2_.

To ensure methodological consistency and allow direct comparison, part of the experimental setup, including selected figures and data, was shared with our previous study involving Osimertinib and Gedatolisib [17]. Figures where data overlap have been explicitly referenced and are intended for comparative interpretation only.

### 4.2. HBMEC Monolayer Permeability and Integrity

To predict the permeability of PKI-587 and LASSBio-1971 through the BBB, we performed an HBMEC monolayer assay, as previously described [59]. Briefly, 8 × 104 cells/well were seeded in 24-well plate inserts (0.4 µm pore size) (Corning Costar Corp., Wilkes Barre, PA, USA), which had been pre-incubated with rat tail type I collagen (4 mg/mL; Sigma, St. Louis, MO, USA) for 3 h. After the formation of an HBMEC monolayer (48 h incubation), the integrity and transport procedures were assessed by measuring the transendothelial electrical resistance (TEER) using a Millicell ERS-2 volt-ohmmeter (Merck, Darmstadt, Germany). TEER was measured before, 30 min, and 120 min after incubation with the respective inhibitors at a concentration of 25 µM. Additionally, the integrity of the HBMEC monolayer was evaluated using sodium fluorescein after 30 min to assess paracellular permeability. For this, a 10 mg/mL sodium fluorescein solution in PBS was added to the insert, and after 30 min, the optical density at 525 nm was measured in the lower chamber (well).

To evaluate the LASSBio-1971 and PKI-587 permeability, 25 µM from each inhibitor was added to the insert (blood), and then the well (brain) was collected at 30 and 120 min and centrifuged at 500× *g* for 5 min to remove cellular debris. The solution was deproteinized by adding cold acetonitrile at a 1:3 ratio. The identification and quantification of PKI-587 and LASSBio-1971 were performed using high-performance liquid chromatography (HPLC). Vincristine (25 µM) was used as a negative control and caffeine (25 µM) as a positive control. DMSO at 2% was used to normalize the data. This concentration was used throughout all experiments.

### 4.3. MTT Assay

Metabolically active cells were assessed using the 3-(4,5-dimethylthiazol-2-yl)-2,5-diphenyl tetrazolium bromide (MTT) reduction colorimetric assay. GB cells were plated in 96 multi-well plates (for 24 h: 4 × 105 cell/mL for GBM02, GBM03, and GBM95, or 5 × 105 cell/mL for A172 and T98G; for 72 h: 2 × 105 cell/mL for GBM02, GBM03, and GBM95, or 5 × 105 cell/mL for A172 and T98G) and then were incubated with PKI-587 (50/10/1/0.1/0.01/0.001 mM) and, LASSBio-1971 (100/50/10/1/0.1/0.01 mM for 24 h, and 50/10/1/0.1/0.01 mM for 72 h). The absorbance was read in a microplate reader at 570 nm, and the cytotoxic concentration for 50% from maximum effect (CC50) was calculated using the Prism 7 statistical software (GraphPad Software, La Jolla, CA, USA). The synergistic effect between LASSBio-1971 and PKI-587 was based on MTT assay, considering the Chou–-Talalay method (Chou 2010). Results of drug combination were obtained by CompuSyn version 1.0 (ComboSyn, Paramus, NJ, USA), and expressed in the combination index (CI) versus Fraction Affected (Fa) graph. Combinations with CI values lower than 1 are considered Synergistic [19]. The selectivity index was determined following Chao [60], dividing the CC50 of the selected drug in the GB cell line by the CC50 of the same drug in a human astrocyte cell. The LASSBio-1971 and PKI-587 concentrations for polytherapy were chosen based on their lower CI and higher Fa. For monotherapies, we used the CC50.

### 4.4. ATP Quantification

ATP measurement was performed using luminescent ATP detection (Abcam 113849) following the manufacturer’s instructions. Cells and isolated or combined drugs were incubated for 72 h, at the following combinations: GBM02 (7 µM LASSBio-1971 and 0.093 µM PKI-587), GBM95 (3 µM LASSBio-1971 and 0.0155 µM PKI-587), and T98G (1,75 µM LASSBio-1971 and 0.186 µM PKI-587). TMZ (500 µM) was used as a control.

### 4.5. Cell Migration by Scratch Wound Assay

Cell migration was performed according to the method described by Liang [61]. Briefly, the GBM cell monolayer was scraped in a straight line with a p200 pipette tip. The debris was removed by washing the cells with phosphate buffer saine (PBS). After, DMEM supplemented with 10 µM Ara-C (without FBS for GBM95 or 2.5% FBS for T98G) was added. Ara-C was used as a proliferation inhibitor. Then, 3 µM of LASSBio-1971 and 0.0155 µM of PKI-587 were added to the GBM95 cell line, alone or combined. For the T98G cell line, the selected concentration was 1.75 µM of LASSBio-1971 and 0.186 µM of PKI-587. ImageJ software (version 1.54p) (National Institutes of Health) was used to record the coordinates for each scratch location using a computer-controlled stage. The scratch width at 24 h was compared to the original scratch width (0 h).

### 4.6. Apoptosis Assay

To evaluate apoptosis, 2 × 106 cells were seeded and incubated for 24 h. Then, 7 µM of LASSBio-1971 and 0.0155 µM of PKI-587 were added to the GBM95 cell line, alone and combined. For the T98G cell line, the selected combination was 1,75 µM of LASSBio-1971 and 0.186 µM of PKI-587. After 24 h, 1 × 106 cells were pellet down (5 min/500× *g*) and resuspended in 100 µL of Binding Buffer 1X. Next, 5 µL of Annexin V (AnxV) and 5 µL of propidium iodide (PI) were added to the cell suspension, and the incubation was performed at room temperature for 15 min in the dark. Then we added 400 µL of binding buffer 1X and proceeded to the CytoflexS (Beckman Coulter) cytometer analysis.

### 4.7. Cell Cycle Analysis

The cell cycle was analyzed by PI incorporation. Briefly, GBM95 cell lines were seeded at a density of 2 × 106 cells/well and incubated for 24 h with 3 µM of LASSBio-1971 and 0.0155 µM of PKI-587, alone or combined. Then, 2 × 106 cells were pellet down (5 min/500× *g*) and resuspended in 3 mL of cold 70% ethanol, drop-wise. After 1 h at 4 °C, the cells were centrifuged and washed with PBS twice (10 min/700× *g*). A total ofTo the final pellet, was added 50 µg of PI and 1 µg RNAse A, in PBS (1 mL final volume), was added to the final pellet, and then it was, incubated for 4 h at 4 °C. The PI incorporation was measured in the CytoflexS (Beckman Coulter) cytometer.

### 4.8. Statistical Analysis

Data (presented as mean + SEM throughout) were analyzed, and multiple comparisons were done performed by ANOVA using Bonferroni’s corrections, with Windows-supported SigmaPlot version 11.0 (Erkrath). Pairwise comparisons were done performed with two-tailed *t*-tests (separate variances). *p* < 0.05 was considered significant. The CI values were calculated using CompuSyn version 1.0. All experiments were performed in at least three independent biological replicates, each consisting of duplicate technical samples. The number of replicates (n) is indicated in the figure legends. Potency values were calculated using the Prism 7 statistical software (GraphPad Software, USA).

## Figures and Tables

**Table 1:** Their cytotoxicity concentration measured the cytotoxic potency of LASSBio-1971 and PKI-587 at 50% (CC50) in a 24 h and 72 h MTT assay. OD was measured at 570 nm, and data were normalized by non-treated control (usual media + 1%DMSO). CC50 and Emax were calculated by concentration–response curves for the target compounds in all GB cells after 24 and 72 h incubation. Values are means ± SEM of three independent experiments in triplicate. This table includes data previously published in [17], reused here for comparative purposes.

	24 h		72 h		72 h
PKI-587	%E_máx_	CC50 (μM)	%E_máx_	CC50 (μM)	SI
GBM 02	57.7	54.2_(22.1–534.7)_	96.7	0.14_(0.03–0.6)_	200
GBM03	87.4	23.1_(14.6–38.35)_	95.5	0.08_(0.02–0.4)_	329.4
GBM95	82.2	2.1_(0.63–8.4)_	99.7	0.03_(0.01–0.1)_	933.3
T98G	98.4	5.1_(1.45–14.34)_	96.8	0.20_(0.11–0.4)_	140
A172	92.4	2.8_(0.53–14.22)_	98.5	0.55_(0.2–1.5)_	51
hASTR	-	-	53	28	-
LASSBio 1971	%E_máx_	CC50 (μM)	%E_máx_	CC50 (μM)	SI
GBM 02	64.3	69.1_(56.1–86.9)_	89.4	16.1_(7.9–22.8)_	3.25
GBM03	70.3	43.7_(39.6–48.2)_	85.4	17.1_(12–24.2)_	3.05
GBM95	67.8	59_(48.4–71.7)_	99.7	9.6_(7.8–10.8)_	5.45
T98G	85.6	35_(24.9–46.83)_	91	2.3_(8.12–16.9)_	4.25
A172	59	56.2_(40.6–84.72)_	87.2	15_(10.1–21.2)_	3.49
hASTR	-	-	75	52.3	-

## Data Availability

Data available under plausible request.

## Supplementary Materials

The following supporting information can be downloaded at: https://www.mdpi.com/article/10.3390/ijms26136392/s1.

## Abbreviations

The following abbreviations are used:

AKT	Protein Kinase B
AnxV	Annexin V
ASTRh	Human Astrocyte Cells
BBB	Blood–Brain Barrier
CC50	Cytotoxic Concentration 50%
CI	Combination Index
DMEM	Dulbecco’s Modified Eagle’s Medium
EGFR	Epidermal Growth Factor Receptor
Emax	Maximum Effect
EMT	Epithelial–Mesenchymal Transition
ERK	Extracellular Signal-Regulated Kinases
Fa	Fraction Affected
FBS	Fetal Bovine Serum
GB	Glioblastoma
GSK3β	Glycogen Synthase Kinase 3
HPLC	High-Performance Liquid Chromatography
IDH	Isocitrate Dehydrogenase
MAPK	Mitogen-Activated Protein Kinase
MGMT	O-6-Methylguanine-DNA Methyltransferase
mTOR	Mammalian Target Of Rapamycin
MTT	3-(4,5-Dimethylthiazol-2-Yl)-2,5-Diphenyl Tetrazolium Bromide
PBS	Phosphate Buffer Saline
PI	Propidium Iodide
PI3K	Phosphatidylinositol-3-Kinase
PTEN	Phosphatase And Tensin Homolog
SI	Selective Index
TEER	Transendothelial Electrical Resistance
TMZ	Temozolomide
