# Supplementary material for: A Pharmacologic Approach Against Glioblastoma—A Synergistic Combination of a Quinoxaline-Based and a PI3K/mTOR Dual Inhibitor
Source: Int J Mol Sci. 2025 Jul 2;26(13):6392. doi: 10.3390/ijms26136392 (PMC12249934; doi:10.3390/ijms26136392)
Supplement: Supplementary file 1 [file ijms-26-06392-s001.zip › ijms-3649822-supplementary.pdf]

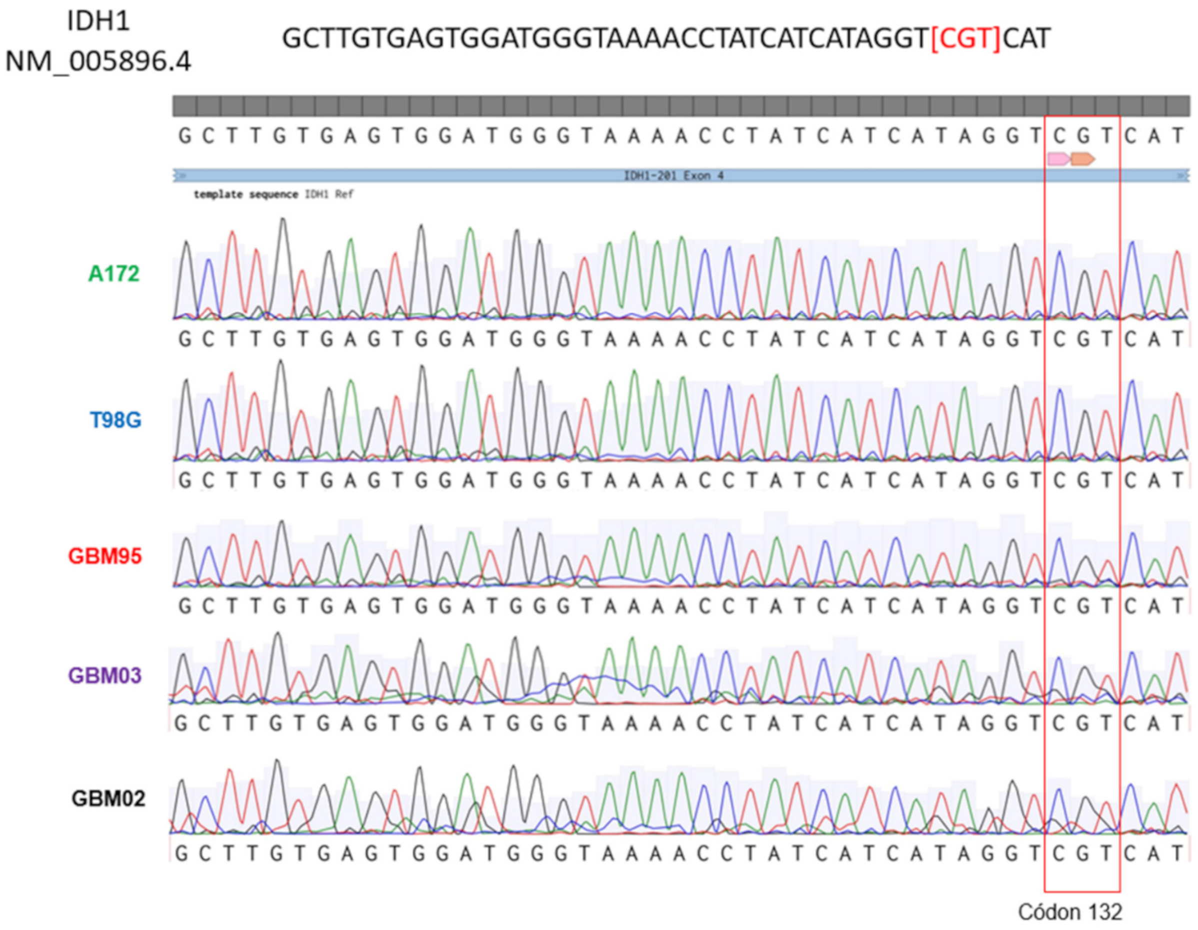


**Supplementary Figure S1 – Characterization of IDH1(Codon 132) genotype of GBM02, GBM03, GBM95, T98G and A172 GB cell lines.**

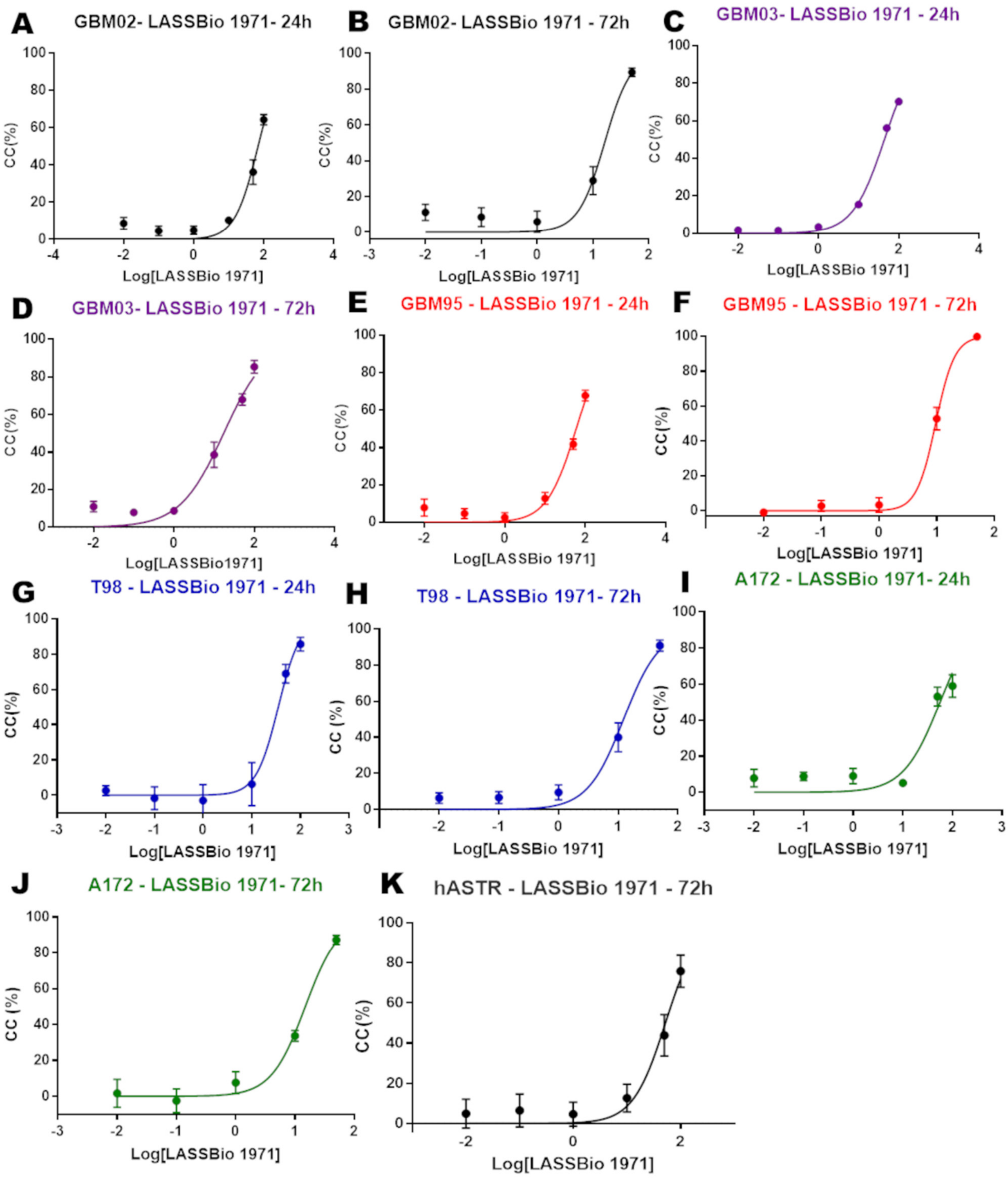


**Supplementary Figure S2 – Related to Table 1. Potency and efficacy of LASSBio-1971 at 24h and 72h against GBM02 (A,B), GBM03 (C,D), GBM95 (E,F), T98G (G,H), A172 (I,J) GB lineages and human astrocytes (K).** Values are means ± SEM of three independent experiments in triplicate.
